# The Foreign Journals: Pathology, Practical Medicine, and Therapeutics

**Published:** 1841-01

**Authors:** 


### PATHOLOGY, PRACTICAL MEDICINE, AND THERAPEUTICS.

### *Development of Acute Glanders by the Introduction of Pus into the Veins of a Horse.* By M. Renault.

M. Renault presented to the Academy of Medicine some anatomical specimens taken from a horse in which acute glanders was developed by the introduction of pus into its veins. The pus was taken from an abscess on the breast of a horse four years old, perfectly free from any glanders or farcy. The animal experimented upon was a small horse of a rustic breed, lean, but in good health, high spirited, and of good appetite. An emulsion was made with four centilitres of pus in ten decilitres of distilled water. This emulsion filtered through fine linen was introduced into the veins of the horse on the 13th of July. The next day a very intense fever manifested itself; the heart beat 90 in the minute; three days afterwards a pustule appeared in the left nostril, and a pustular eruption showed itself on the chest; on the following days the acute glanders was shown with all its characters. The animal died on the 20th of July.

On a post-mortem examination there appeared ulcerated pustules on the surface of the pituitary membrane, and purulent infiltrations in its substance; in the lungs there were many metastatic abscesses and lobular pneumonia; and in the spleen and walls of the heart purulent granulations were found.

*Bulletin de l'Académie Royale de Médecine.* 15 *et* 31 *Août,* 1840.

---

### *Irritation of a Branch of the Trigeminus Nerve at its central extremity.* By Dr. Budge, of Altenkirchen.

A peasant woman, forty-six years old, was affected for the first time in her sixth year with a severe periodical pain over the right eye, for which she could ascribe no other cause than exposure to cold and damp one winter's day. Previously she had always been healthy, and her parents attained a very advanced age. The pain continued from her sixth to her twelfth year, recurring about every fortnight, lasting from twenty-four to thirty hours each time, and then leaving her again well and hearty. When she was twelve years old it ceased; and during the next twelve years never once returned. In the interval she commenced and regularly continued menstruating. But at the end of this time, when twenty-four years old, she had a child, and eight days after delivery her old pain returned; from that time, now twenty-two years ago, she has never been a week without it.

The pain usually begins at midnight, awakening the patient from sleep, and lasts to the same time in the second night following, i. e. for about forty-eight hours. It is preceded and accompanied by chilliness, which at last terminating in sweat, indicates the approach of the end of the attack. The pain extends from the lamdoidal suture of the right side, and is most severe at the posterior and anterior surfaces of the upper eyelid, about half an inch above the brow, and a short distance below the eye of the same side. It is a stabbing pain, and so intense that the mere thought of it fills the patient with horror; it continues uninterruptedly through its whole period, and shortly before it ceases attains an indescribable severity. The slightest touch excites intense agony in the affected part. The immediate neighbourhood and all the rest of the face are as to sensation perfectly natural; but the features on the right side are less marked than on the left side of the face. The cornea (during the pain) is rather turbid, and the eye is filled with tears; the sclerotica is reddened, and the vessels of the conjunctiva turgid; the tarsi are also swollen and red. The whole eye is turned outwards, so that the cornea is nearly hidden; and the patient can with difficulty look straightforward with it, partly from loss of power over it, and partly because the straight position, if retained but for a few instants, excites giddiness and vomiting.

Soon after the commencement of the pain, giddiness comes on, and compels the patient to keep her bed; it increases with the pain, and usually lasts a day longer than it does. The patient is at the same time constantly tormented by sickness and vomiting of food, mucus, and bile. Her tongue is quite clean and her bowels regular, but she has no appetite. The organs of the abdomen and chest and the pulse are in a normal condition.

The first and second cervical vertebræ are a little sensitive on pressure, and from the third to the second dorsal there is extreme sensibility, especially over the third, fourth, fifth, and sixth cervical. As often as these are struck the patient flinches and complains of pain in the right eye, and immediately after vomits. The right arm also, which sometimes moves spontaneously when the pain is very intense, is sometimes convulsed when these vertebræ are pressed.

When the paroxysm is about to cease, the intensity of the pain, the squinting, and the giddiness decrease, and soon after a general sweat comes on. At the last the patient sneezes once or twice (and always, she says, through the right nostril), and then, except for the giddiness, which lasts a day longer, the disease is for the time removed. Every fresh attack, however, is severer than the preceding, in respect both of the pain and of the squinting. The paroxysms are rarer in the summer than in the winter and spring; mental anxiety and passion accelerate and aggravate them, but neither menstruation nor pregnancy has any influence on them.

In the intervals between the attacks of pain, there is no external appearance of illness whatever, except a slight outward squint; and, but for the influence of pressure on the spine, the woman might be deemed perfectly healthy. By this, however, though there is never any spontaneous pain in the back, all the signs of the disease may be in a slight degree voluntarily reproduced; as soon as the lower cervical vertebræ are pressed, the patient complains of the pain of her eye and head, the right eye turns outwards, tears flow into it, and sickness or even vomiting comes on. And again, all these phenomena cease as soon as the pressure is remitted.

Of the above singular symptoms, the author offers the following explanations:

The first and most important, the pain over the right eye, is evidently the result of a state of irritation of the majority of the fibres of the frontal branch of the trigeminus, whose exact course it follows; for the patient says that the pain is not in the eye itself, but passes above it from behind forwards, and extends upwards and a little inwards towards the nose. The pain in the back further shows that the exact seat of this irritation is the central extremity of the nerve, of which, as is well known, the roots have been traced to the region of the third and fourth cervical vertebræ, and probably go down still lower. Immediately over this part, the patient has pain on pressure: and by it the pain in the eye may be produced and increased at will, although, except during the paroxysm, no more pain is excited by pressure on the right than by that on the left eye.

The pain in the other adjacent vertebræ probably results both from the stimulus of pressure on the extremities of the nerves over them, which from their proximity to the nervous centre are especially liable to exalted excitability, and from the immediate effects of the pressure on the membranes which are themselves excited and sensitive in an unnatural degree.

The drawing of the eye outwards is the result of an influence on the sixth nerve; an influence reflected from the sensitive nerves. It is not probable that the central ends of the fibres of the sixth are directly irritated; for if that were the case, the same extremities of the other nervous fibres that lie between the sixth and the fifth would most likely be also irritated, and the morbid phenomena would be more extensive. The twitchings of the right arm may also in like manner be regarded as reflex motions.

The organic alterations in the eye must be considered as signs of the excitement of the trigeminus; and as when a sensitive nerve is divided, nutrition is materially impaired, and the part it supplies undergoes a kind of maceration, so when one is irritated the same part is inflamed.

The giddiness is thus explicable: fibres of the trigeminus may be traced into the crura cerebelli ad frontem, near the spot from which the nerve comes out, and as Magendie has shown that when these crura are divided, the balance of the body seems to be lost, it is clear how an irritation of the trigeminus induces giddiness, and how that symptom is most marked when the signs of irritation are passing off or gone.

The vomiting is explained partly by the affection of the spinal cord and partly by the influence of the nerves on the sight. (In explanation of this the author refers to his own investigations on vomiting recently published.)

The shivering is the result of the stimulus of the central extremities of the sensitive nerves in the spinal cord; and it is comparable with the results of several of the author's experiments, in which he has observed that on exposing the spinal cord in a dog or other animal, and gently stroking it with a feather, all the characters of a shivering fit are immediately produced. In this, as in all other cases, the condition of the central extremity of the nervous fibre is felt as if it were that of its peripheral extremity.

The sneezing at the end of the paroxysms probably results from the nasal branch of the trigeminus becoming involved in the excitement when the irritation of the frontal is at its greatest height. The excitement is in this case also probably communicated in the nervous centre though felt where the fibres are distributed; and it is communicated not to the sensitive nerves alone, but by reflex action to the nerves of the respiratory muscles.

The author, believing that the essential cause of the symptoms was a congestion of the vessels of the frontal nerve, had ten cupping-glasses applied every eight days near the vertebræ at which pain was produced by pressure; these were applied three times; mercurial ointment was rubbed in morning and evening, and four grains of sulphate of quinine with two of extr. nucis vomicæ were given in two doses every day. Their result was most striking; the patient, who had for twenty-two years been entirely unrelieved by a variety of methods of treatment, had no severe attack after this was employed; after several months had elapsed she continued well, and even the divergence of the right eye was in some degree lessened.

*Casper's Wochenschrift. October* 3-10, 1840.

---

*Effects of Over-eating.* By Dr. Ritter.

A girl, three years old, went with her father to gather plums; as they fell from the trees she kept eating an enormous quantity of them, and then, feeling very thirsty, drank cold water from a spring close by. Soon after returning home, having seemed previously quite well, she suddenly fell down as if struck dead by lightning; her eyes were convulsively pulled about, and she lay quite senseless. The author being immediately sent for found her with her eyes staring wildly, unconscious, insensible, and speechless; her pulse was suppressed, scarcely perceptible, and small, her respiration short and quick, frequently interrupted by sighing, and her face pale. The precordial region was distended and very tense. An emetic, containing one eighth of a drachm of vinum antimonii and one eighth of a grain of tartar emetic, was immediately given, and was repeated every five minutes, but after several doses produced no effect. Three powders were then given at intervals of ten minutes, each of which contained a grain of tartar emetic, and six grains of ipecacuan, but these excited only retching; vomiting was at last brought on by tickling the pharynx with a feather. The materials discharged consisted of a huge mass of tough, half-digested plums with some frothy fluid. The severest convulsions now ensued, especially on the right side of the body. The eyes rolled irregularly and convulsively in their orbits; the mouth was drawn over to the right side, and watery froth kept running from it; the right arm and foot were set into a constant swinging motion, the face was earthy, the glance oblique, and the nose pointed, in short there were all the appearances of closely impending death. Some strong coffee and some wine and water were given, but the convulsions still continued. A mixture of equal parts of castor oil and almond oil was next prescribed, and a spoonful of it was given every ten minutes; clysters, containing oil, vinegar, and salt, were also administered. Soon after the second of these, borborygmi commenced, and were presently followed by the discharge of an enormous mass of half-digested plums, which was again repeated for several times. The child not long after came to herself again, and though weak was still free from apparent danger. She slept well during the night, and next morning, with the exception of dullness and repeated diarrhœa, was well and in good spirits.

*Medicinische Zeitung. October* 14, 1840.

---

*On the Medical Action of the Bezoar of the Lacrymal Fossa of the Deer (Deer's tears) in Nervous Diseases.* By Dr. LÖWENHARDT.

THE substance here described is a moist, strongly-smelling, fatty matter, which appears to the author to be secreted by the mucous membrane lining a fossa just below the anterior canthus of the red deer (*cervus elephas*). It is found in this situation all the year, but in greatest quantity, it is said, during the rutting-season, it appears to serve by the diffusion of its powerful scent to attract or to keep off other kinds of animals. It exists only in the adult individuals of the species.

The smell of this bezoar is peculiar, very penetrating, strongly balsamic, much like that of amber mixed with a little benzoin, only still more pungent, and adhering half or all the day to the substances with which it comes in contact. Its taste is in like manner balsamic, and its most active principle is a peculiar resin which is of a yellow colour and lighter than water. Its analysis yielded resin (with a mixture of etherial oil), a very small quantity of fatty oil, wax, cellular substance, ammonia, and colouring matter, with muriate of soda and phosphate of lime; but it is probable that a part of these constituents are derived from the hair which is usually entangled in it.

It appears, by several cases which the author describes and refers to, to have a certain value in all the diseases in which castor, musk, valerian, and the other allied articles of the materia medica have been hitherto, and often unsuccessfully, employed; as hysteria, epilepsy, difficult menstruation, prolonged hooping-cough, &c. The modes of administration which the author recommends are, 1st, in substance, in doses of from 5 to 10 or 15 grains, twice or three times, in pills with mucilage; and 2dly, in tinctures composed of one part of the bezoar to six parts of rectified spirit, or of etherial spirit in doses of ten, fifteen, or twenty drops twice or three times a day.

*Medicinische Zeitung. August* 12-19, 1840.

---

*Cure of a Severe Gouty Pain by the Electric Ray.* By Dr. A. CHAERETES.

N. suffered three months without any evident cause from a very troublesome gouty pain in the first and second joints of the right fore-finger. He had used all the usual means against it, both internal and external, without benefit; on the contrary, the disease increased so much that he could not sleep during the greater part of the night, and in the day could not hold his pen. Thus he was going on one morning, trying to think of some new remedy by which he might possibly relieve his pain, and carefully avoiding meeting any one, lest an incautious touch might renew it now it was a little assuaged, when a fishing-boat drew near to sell the fish her men had just caught. Among them the patient remarked the electric ray, and being acquainted with its electric properties, he bought it and carried it home alive in a vessel filled with sea-water. Although the fish was but small, yet it possessed a remarkable electric power; and the patient had scarcely touched it with the middle finger of the diseased hand, when he felt a severe shock and a peculiar pain in the elbow, with a numbness of the whole arm and of the fore-finger that had been the seat of the pain. After the lapse of a quarter of an hour, the numbness gradually ceased, and there remained only the peculiar sensation in the elbow, and that in a less degree. The fore-finger still gave indeed severe pain, but only when it was moved. Two hours after a second experiment was performed, of which the results were similar but more violent, and with this difference, that the fore-finger could soon afterwards be voluntarily moved without any pain, and when moved by the other hand, gave not the usual severe suffering, but only an unusual and peculiar sensation. This condition continued without material alteration till seven in the evening. At that time when the patient again repeated the experiment and touched the fish with the fore-finger itself, he felt a slight shock; but when he stimulated it with a piece of iron, he received seven shocks successively, and the fish soon after died.

That night the patient slept quietly; in the second he still felt only a numbness and weakness in the motions of the finger, which were completely removed by cold baths and the cold douche in five days more. Since that time the finger has been quite freely used, and there has not remained the least trace of its former condition.

[Dr. Bouros, by whom this case is communicated, has added some notes respecting the uses which the ancients made of electric fish, but they are not of much interest, and may be found in the Dict. des Sciences Med. *art.* Poisson. He believes the fish used in this case was the Torpedo Narke. There is no reason given for calling it a gouty pain (*Gichtschmerze*) of the finger; was it not rather neuralgic?]

*Casper's Wochenschrift. August* 29, 1840.

---

*On the Uncertainty of the Signs of Peritonitis and on a New Character of that Disease.* By Luigi Sementini.

The chief object of this memoir, is to prove the fact which the author says he has tested by constant observation for upwards of forty years, namely, that in all cases of peritonitis, in whatever part of the abdominal cavity the inflammation is seated, there is pain in the pubes and upon the great trochanters; which if not spontaneously felt is always developed by pressure, and of which the severity is directly proportionate to that of the peritonitis.

This fact, which is said to be confirmed by the clinical observation of others, the author believes is explicable by the relation of the nerves of the parts in which the pain is felt to the peritoneum, and by its connexion with the fasciæ and muscles about them. In addition to its value in the diagnosis of even the most obscure and latent cases of peritonitis, in all of which this sign is present in a degree proportioned to the severity of the disease, the author has found it of value as an indication of treatment, and has obtained great benefit from the application of leeches and blisters over the trochanters instead of on the abdominal walls.

*Annali Universali di Medicina. Settembre,* 1839.

---

*Case of Convulsions of a peculiar kind.* By G. Terrone.

R. S. a country labourer, of sanguine temperament, came to the hospital in March, 1838. He said he had always enjoyed good health, but had been constantly subject to poverty and hard work. Six months ago he began to suffer from a painful contraction of the back, which tormented him from time to time, and was not alleviated by any of the means that he had employed. On examination, the affected part presented a curious appearance. The mass of muscles between the fourth and the eighth dorsal vertebræ, and for two inches width over the corresponding ribs rose up in the form of a compact, resisting, and hard globe; continued so for a few instants, and then disappeared and left the part in its natural condition. Immediately after a similar swelling rose up on the opposite side; and so on, disappearing on one side when it appeared on the other. The diaphragm, and the abdominal and intercostal muscles participated in these convulsions, and they were accompanied by remarkable constriction of the throat, and frequently by slight hiccup. They occurred every five minutes, and when they ceased they left the patient in a perfect state of health.

The reader can form as good an opinion as the author does of the pathology of this affection. It is sufficient to add that it was gradually cured by cupping and the application of tartar-emetic ointment along the dorsal region of the spine, and by warm baths, with the occasional use of sulphuret of antimony and acetate of morphia.

*Annali Clinici dell' Ospedale degl' Incurabili. Ottobre,* 1839.

---

*Apoplexy, with Hemiplegia, in consequence of Fright.* By Dr. Ritter.

The only peculiarity of this case is its cause. A robust and rather plethoric woman, thirty-eight years old, was in perfect health and speaking to a neighbour, when her servant-girl frightened her by brandishing a bright spiral wire over her head, so as to make it look as if a snake were falling on her. In her fright the woman suddenly fell down as in an apoplectic fit, and remained for some time nearly unconscious. When examined she complained of a noise and beating in the left side of her head, deafness of the left ear, and of blindness and loss of taste on the same side. She could not move any part of the left side of the body, and in every respect resembled a patient suffering from hemiplegia in consequence of sanguineous apoplexy. By active antiphlogistic treatment, and various other measures, she was gradually restored from this state in about three months.

*Medicinische Zeitung. September* 9, 1840.

---

*Observations on the Therapeutic Efficacy of Ammoniuret of Copper in Chorea.* By Dr. Fedele di Fiore.

Two cases are related of genuine chorea to prove the value of this once esteemed remedy, which in Italy passes by the name of "the specific of Stissero." In each case nearly all the usual and most active methods of treatment had been adopted before this was resorted to; bleedings, leeches along the spine, anthelmintics, purgatives, cold baths, and various antispasmodics had all signally failed to produce benefit, when the ammoniuret of copper was commenced. In doses beginning at one eighth, and gradually increased to half a grain twice a-day; the latter effected a gradual but perfect cure; in one case within two months, in the other in one month.

*Annali Clinici dell' Ospedale degl' Incurabili. Ottobre,* 1839.

---

*On a Sedative Lotion in Headaches, Congestions, and Cerebral Fevers.* By M. Raspail.

Professor Raspail, in a letter to the editor of *l'Expérience,* gives the mode of preparing a lotion, the sedative effect of which, he says, is almost instantaneous. It is as follows:

| | |
|---|---|
| Liquor of ammonia (Qy. the strength ?) . . | 100 parts |
| Distilled water . . . . . . . . | 900 ,, |
| Purified marine salt . . . . . . . | 20 ,, |
| Camphor . . . . . . . . . . . | 2 ,, |

Essence of rose or some other scent, in the necessary proportion. The whole dissolved cold.

A piece of linen is to be steeped in this solution and applied over the part of the head that the patient points out as the seat of pain, taking care, if it is on the forehead, to apply a thick bandage over the eyebrows, to prevent any drops of the fluid passing into the eyes.

M. Raspail says he has seen headaches intolerably violent, accompanied by photophobia, and retraction of the globes of the eye, disappear completely, from a quarter to half an hour after the application of one wetted cloth. The linen is to be soaked as often as a new access of pain is threatened, and left on the head until it is necessary to soak it anew. In the numerous trials the author has made with this solution, first on himself and afterwards on others, he has been struck by two circumstances of interest in connexion with organic chemistry, and symptomatology. When in a violent attack of cerebral fever, we apply on the principal seat of the inflammation a concentrated solution of marine salt, an evident odour of chlorine is disengaged, the diseased reaction being analogous to the decomposing and deoxygenating action of manganese, in the elimination of chlorine from marine salt, by means of sulphuric acid. Is this sign constant in affections of this class? It is for experience to decide. When on the contrary we employ a solution of ammonia, a strongly characterized *hircine* (goatish) odour is manifested. The same odour has been disengaged on the application of hydrochloric acid to the skin. M. Raspail has drawn the attention of the profession to this subject in order that they may employ this formula, and fix their attention on the analyses of the disengaged substances, as they may become characteristic of special affections.

*L'Expérience.* 24 *Juillet,* 1840.

---

*Case of Posthumous Variola.* By Dr. CHANSAREL.

A GIRL, aged five years and eight months, who had been vaccinated, was suddenly attacked, when in perfect health, with a varioloid eruption and fever. About twelve hours afterwards the redness of the skin and the pimples totally disappeared, cerebral phenomena supervened, and death twenty-six hours after the first appearance of illness. On the next day, thirteen hours after death, "I saw," says M. Chansarel, "to my great astonishment, a great number of pimples which had appeared after death, and which were much more developed than those I had seen during the life of the child. They were seated on the face, neck, chest, and buttocks; those in the latter region being larger, equalling the size of a lentil. They had the aspect of variolous pustules, surrounded by a red areola, depressed in their centre, and contained a small quantity of fluid. The body exhaled a fetid odour, and exhibited some red and livid spots, putrefaction not having been so slow as it appeared."

*L'Expérience.* 27 *Août,* 1840.

*Extracted from Bulletin Medical du Midi. Juin, Juillet,* 1840.

---

*Mode of Resolving Engorgements of the Spleen.* By M. VOISIN, of Limoges.

THE author has employed the following treatment with success in eight or ten cases of these engorgements after intermittent fevers. In three or four of these cases the diseased organ occupied about two thirds of the left half of the abdomen. The treatment is simply to apply a mercurial plaster (*vigo cum mercurio*) with which is incorporated six or eight scruples of the sulphate of quinine, more or less. It is to be renewed when the matter of which it is composed is exhausted, that is, from forty to fifty days. The advantages of this mode of treatment are: 1. It saves the patient from the disgust which he undergoes when the quinine is administered by the mouth. 2. The absorption and consequent action of the remedy are continued. 3. This absorption and this action are accomplished in the immediate neighbourhood of the diseased organ. 4. Owing to the continuance, the fever does not reappear.

This treatment has alone sufficed for the cure. The period of cure will vary as the spleen is more or less engorged, the patient more or less aged, and the skin more or less readily absorbing. Ordinarily, two or three months suffice.

*Gazette Medicale de Paris.* 12 *Septembre,* 1840.

*On the Utility of Graduated Compression of the Abdomen by Bandages, in the Treatment of Ascites.* By Professor L. MORELLI, of Siena.

FROM among many cases that were either cured or materially relieved by this mode of treatment, the author relates one in which its benefit was most strikingly shown. It was that of a woman upwards of fifty years old, who from poverty, and other sources of misery, had become gradually more and more ill, and had at length come to the Clinical Hospital with ascites from diseased liver. She had been salivated, and had undergone a variety of treatment, and had been tapped four times. The author recommended his system of bandaging, but for a long time the patient refused to adopt it, and in the interval it was found necessary to perform paracentesis eight times more. After the twelfth operation the patient, now reduced to a most perilous state, agreed to submit to the bandaging, of which she had seen the good effects in a dropsical child. The best kind of bandage consists of a band extending from the lower third of the sternum to the pubes, and round to the loins, and there gradually tightened by means of strips of leather, with several holes in them for the passage of the points of small pins. The whole is prevented from shifting upwards by two strips of strong cloth which pass downwards, and are stitched behind in the neighbourhood of the sacro-iliac symphyses. This apparatus being put on, and gradually tightened, the abdomen of the patient slowly returned to its natural size; the urine flowed abundantly, her appetite and sleep returned, and, at length, after nearly three years' illness, in which she had used a variety of medicines, and had been tapped twelve times, she was restored to good health. A year afterwards, having discontinued the bandage, the ascites appeared again in a slight degree, but purgative medicines and the reapplication of the compression again speedily removed it.

At its first application, and for some days afterwards, the bandage produces great inconvenience; so much, that many patients refuse altogether to wear it for a sufficient length of time; but by a proper selection of cases, and careful management of them in other respects, the author asserts that compression will be found one of the most successful means that have ever been employed against ascites. It must be confessed, however, that he gives no indications, nor does he himself seem to have learned in which of the very different circumstances in which ascites occurs, his method is either applicable or likely to prove useful.

*Annali Universali di Medicina. Marzo,* 1840.

---

*On Hydrophobia and the relation between the Retina and the Respiratory Nerves in that Disease.* By Dr. A. TRIBERTI, Physician in Ordinary to the *Ospedale Maggiore* of Milan.

THE main object of this paper is to recommend that hydrophobic patients should be kept in almost perfect darkness, and carefully excluded from the view of anything bright or glistening, to avoid the severe paroxysms which the sight of such objects has never failed to bring on in all the cases that the author has seen. These have been seventeen in upwards of thirty years' practice. In all these cases the hydrophobia did not once come on before the 25th, nor after the 60th day from the reception of the bite. All were hydrophobic when they were brought to the hospital, and in all, the wound which had cicatrized a few days after the bite, was found red, with heat, burning, and a painful and spasmodic sensation of the whole limb, or of all the bitten part. In five of the seventeen cases the wound had opened again, and presented a malignant ulcer, discharging a fetid serous fluid. All died on the second or on the third day from the commencement of the attack. All complained of languor and lassitude, which were accompanied by anxiety and deep sighing; all were desirous of being left alone and in the dark; in all the pulse was quick and irregular, and in some rather full. After the first twenty-four hours of their being in the hospital, the patients all complained of tightness and oppression of the præcordia, with dyspnœa and excessive thirst, which they could not alleviate by drinking fluids, though they could swallow small pieces of solid food. The spasms of the muscles of the pharynx produced in all the patients a distressing sensation of a ball or globe in it, which was always increased at the sight of any drink, but was diminished by swallowing any solid substance. The spasm of these muscles was also in all cases accompanied by convulsive movements of the head, and by severe tremblings of the other muscles of the neck.

*Annali Universali di Medicina. Giugno,* 1840.

---

*On a stony Concretion in a Human Nose* By Stefano Grandoni.

A woman of the province of Brescia, aged 32, was brought to the hospital for a disease of six months' standing, consisting of a calculus, which was situated in the left nostril. Of this affection she was entirely cured by the extraction of the calculus, which was effected with a pair of polypus-forceps. The concretion, in the condition into which the operation had reduced it, was in fragments of unequal sizes, covered with small rough prominences, furrowed longitudinally, and invested by a kind of crust inseparably united to its chief mass, slightly tinged of an iron-rust colour, and beset with minute pits. The fracture of this substance showed that the texture of the whole mass was very similar to that of the compact calcareous earth, in which there is some crystalline glittering. The concretion had a specific gravity of 1·4, and therefore sank in water; at the ordinary temperature it did not emit any odour; it weighed altogether seventy-six grains. In none of the fragments could there be recognized the nucleus of the concretion, unless one could regard as such a little seed of some grass which occupied the centre of the largest fragment. On being subjected to an accurate chemical analysis (the details of which are given), a hundred parts of the calculus yielded the following constituents:

| | |
|---|---|
| Phosphate of lime . . . . . . . | 55 |
| Carbonate of lime . . . . . . . | 18 |
| Carbonate of magnesia . . . . . . | 7 |
| Organic matter, with traces of iron . . | 20 |
| | 100 |

*Commentarii dell' Ateneo di Brescia,* 1837-8; and *Annali Universali di Medicina. October,* 1839.

---

*Angina Pectoris, terminating fatally by Rupture of the right Coronary Artery.* By Dr. A. Gallardi.

A shoemaker, aged about 40, was received into the Milan Hospital, May 21, 1834. He was of good constitution, well formed, of rather nervous temperament, and somewhat addicted to strong drinks. He said that for some months past he had at times, while at work, felt some difficulty of breathing, with a loss of power in the left arm, and subsequent slight lipothymia. Twice in the last month, on going out of his cottage into the open air, he had felt severe pain in the heart, extending to the left scapula and arm, and accompanied by a momentary reeling and slight obscuring of the senses. These symptoms had occurred with unusual severity two days before his admission, after drinking some spirits, and were followed by a death-like syncope; from which, however, he was quickly and completely restored by a large bleeding.

On the day of his admission the patient was found suffering from severe dyspnœa, of a character which led the author at once to suspect a disease of the heart, although it was combined with a number of anomalous symptoms that could only be referred to hypochondriasis. On percussion, both sides of the chest had a natural resonance; but in the median line and the adjacent part of the left side, there was dullness over even a greater extent than in most cases of hypertrophy with hydropericardium. The movements of the heart were scarcely perceptible to the hand placed on the cardiac region, but posteriorly they were even more sensible than natural. The pulse was in no degree intermitting, but small, sometimes very frequent, at others quick, and very hard. The patient was bled to twelve ounces and had other antiphlogistic remedies, and on the following day was much better. On the 23d, however, all his symptoms were worse than ever; he had excessive dyspnœa, severe pain in the heart, constant dry cough, and frequent faintings. The heart could be only very obscurely felt in front, but close by the vertebral column its action was more than ever distinct; the pulse was still very small and hard, and the left hand and forearm were œdematous. The patient was repeatedly bled and subjected to severe antiphlogistic treatment, but all his symptoms continued or grew worse; the œdema spread gradually over the whole body, and on the 31st he died suddenly in a fit of syncope.

The body was examined thirty hours after death. The contents of the skull presented nothing unusual. The pleural cavities contained a large quantity of serous fluid; the lungs were both œdematous, and their lower lobes were much compressed both by the fluid and the immensely dilated pericardium. This sac was evidently distended by some dense consistent fluid containing small grumous masses, and pressing the heart unnaturally far backwards. On cutting it open along its anterior wall, a large quantity of grumous blood and serum flowed from the pericardium; and "at the aperture thus made there soon appeared a body whose surface was of a whitish colour, beset with innumerable uniform oblong appendices, which might remind one of the surface of the villous heart, as described by pathological anatomists. This pseudo-organism, which was of a conical form, was fixed on the heart, and adhered by its base to the base of the ventricle and the right auricle: it was as large as the heart itself. On washing the parts, it was found that the base of this tumour adhered to the parts just mentioned by false membranes, and that it had obtained by them an adhesion to the right coronary artery, in which there was a laceration about two lines in length with elevated edges;" through which the blood had been poured into the pericardium, and, becoming organized, had been developed into this remarkable tumour. At the left side the adhesions by which the tumour had before been attached to the heart had been broken down, and thus the exit of the additional quantity of blood which had been found in the pericardium had been permitted. The heart itself, and its valves, were perfectly healthy, but the aorta and its great branches presented signs of arteriasis, and there was a cartilaginous and osseous elevation close by the origins of the coronary arteries: the pulmonary artery and its branches were similarly diseased. The contents of the abdomen were generally healthy.

To this case, which we believe is unique, the author has added very lengthened observations, of which, however, those only are sufficiently important which refer to the probability that the rupture of the coronary artery and the first effusion of blood took place when the patient was seized with the very severe symptoms two days before his admission, and that the second and fatal effusion occurred at the instant of the fainting fit, in which the patient's life was terminated. It is to be regretted that the general condition of the coronary vessels is not mentioned.

*Annali Universali di Medicina. Decembre,* 1839.

---

*On Tubercles of the Larynx and Fauces.* By Dr. POPKEN, of Jever.

THE disease to which I have given this name, at the risk of its being objected that it does not consist of true tubercles, but only of diseased mucous follicles, has often presented itself to me since my attention was first drawn to it, and exhibits so much that is peculiar in its phenomena and course that I cannot refer it precisely to any of the generally known chronic diseases of the larynx. I have never seen it before puberty, nor in advanced age, but always in young subjects, and chiefly in males; neither could I ever trace any connexion between this and any constitutional chronic diseases, as syphilis or scrofula. I have so rarely found it coincident with other local affections, and especially with those of the lungs, that, in a doubtful diagnosis, I rather regard this disease in the throat as a sign that there is *not* a vomica in the lungs.

In general the patient, in whom this peculiar alteration of the mucous membrane lining the fauces and larynx exists, begins, without any evident cause, to cough and hawk very frequently: but he complains of no pain in the affected part, and is usually in perfectly good general health, so that the whole disorder appears like an obstinate cold, and is annoying only from the incessant irritation that excites the hawking. The disease may thus continue apparently without alteration for weeks or months, till at last (and usually in the morning after drinking) the ejection of one or more small masses of an elastic matter, just like chalcedony in colour and semitransparency, attracts the patient's attention, and, though followed by temporary relief, makes him anxious.

If the fauces be examined at this period of the disease, one finds the whole of their posterior wall thickly beset with small, round, bright red, semitransparent bodies, varying in size from that of a pin's head to that of a pea, and just like the granulations which Laennec and Louis describe as the commencement of tubercles. On some of these granulations one sees adhering firmly to them the opaline mucous globules already mentioned, and just similar in size and form to the bodies beneath them. This granulated condition of the mucous membrane, which, as far as I know, has not been before remarked by any one, extends down as far as the eye can reach, and is always a certain sign of the existence of similar tubercles in the mucous membrane of the larynx, and of threatening laryngeal phthisis; for, although the latter disease does not necessarily, and still less rapidly follow, yet instances of its occurrence, as a signal of the milder affection just described, are not wanting.

This fatal result of the disease, however, may, the author asserts, be always averted by early and judicious treatment. The means which he has employed with the best success are a nutritious, but not stimulating diet, constant warmth round the throat, and the administration of aromatic and tonic medicines, such as ammoniacum and myrrh, or myrrh, extract of bark, and sulphate of iron.

[The disease described by the author must be familiar to every practitioner, but we are not aware that the anatomical condition on which it depends has been before pointed out, though it is distantly alluded to by Henle (Ueber Schleim-und Eiterbildung, in Hufeland's Journal), who found the expectorated substance to consist of morbidly altered epithelium cells. The name which the author has given it is most objectionable, expressing only what the disease is *not;* it is an affection of the mucous membrane, exactly analogous to acne of the skin, and its name should express, if possible, that it has its origin in disease of the mucus-glands.]

*Casper's Wochenschrift. August* 22, 1840.

---

*Dropsy after Scarlatina, unaccompanied with Albuminous Urine.*
By Dr. Philipp, of Berlin.

During the spring of the year 1840, scarlet fever was very prevalent in Berlin, especially in the northern and eastern parts of the city. A hundred cases of the disease came under Dr. Philipp's notice, who found that it resembled the late London epidemic, in being invariably followed by dropsy. Some cases of the fever, indeed, terminated fatally from head affection; in other, and more numerous instances, the patients died from putrid sore throat; but whenever that was not the case, dropsical symptoms, more or less severe, occurred from the twelfth day up to the fourth or fifth week after desquamation had commenced. In some children, too, dropsy came on without being preceded by any symptoms whatever of illness.

There were two points in which the Berlin epidemic differed from that which prevailed in London, one was, that, instead of being a fatal disease, not one child died; the other, that, although the author tested the urine in sixty cases, he never detected the presence of albumen by heat alone, and it was only in a few cases that nitric acid gave signs of its presence.

He states, as in some measure accounting for this, that albuminous urine in dropsy is very rare in Berlin; for, during two years in which he has been physician to the poor in one district of the city, although he has had 150 new cases in a month, he has met with but two instances of Bright's kidney.

*Casper's Wochenschrift. Aug.* 29, 1840.

---

*Case of Idiopathic Lymphadenitis.* By Dr. Pogliaghi, of Milan.

C. C. aged forty-eight, a man of robust constitution, but of very debauched habits, after a few days' illness, was attacked by a troublesome inflammation, which appeared first at the sides of the face, extending from the front of the ears along the margins of the lower jaw, and meeting together at the chin. Emollients were used without benefit, and fever coming on, a physician purged and bled him twice. Being in some measure relieved by these means, the patient neglected himself and suffered the disease to make slow though scarcely perceptible progress. After some days of apparent calm, small almost painless tumours appeared at the lower part of the neck; to these succeeded others in the axillæ and the groins, and after these, fresh ones in the hams and at the bends of the elbows; all of which in the course of three days had increased so as to render the movements of the joints inconvenient. The patient now resorted to various quack medicines, and at last growing worse was sent to the infirmary, having been ill altogether about seven weeks.

At first sight it was supposed that he had the disease known by the name of *orecchioni* or of parotiditis rheumatica, for his face presented at either side a slightly painful swelling, commencing in front of the meatus auditorius externus, following the lower margin of the jaw, and meeting that from the opposite side at the chin; and feeling as if it were formed by an agglomeration of divers lobes. The further examination found some tumours as large as pigeons' eggs at the base of the neck, others much larger in the axillæ, others as large as those in the neck at the elbows, and again others as large as a strong man's fist in the groins, and of various sizes in the hams. The tumours on the left side were generally larger than those on the right; the skin covering them all had its natural colour, and there was no increase of heat. Motion of the joints was painful. The patient had slight fever and rather a full pulse; his face was livid red, and his eyes prominent as if he had a ligature on his neck; his head was heavy, his tongue coated, his appetite not destroyed, his respiration free but accompanied by frequent sighing, his abdomen presenting nothing morbid.

The patient was bled nine times (for reasons which are given in detail), leeches were twice applied to the neck, and mild purgatives, followed by pills containing calomel and extract of aconite, were given. After the first bleeding, (from which as from all the rest the blood was buffed), the patient seemed somewhat relieved, and the tumours appeared less hard; but subsequently the fever became more severe and had regular evening exacerbations, and the patient had frequent attacks of dyspnœa. At first these were slight and occurred most frequently in the night, but afterwards they become more severe and prolonged, and in one of them he died suddenly, nine days after his admission into the hospital, and about two months from the commencement of his illness.

At the examination of the body, some of the enlarged glands were found in a state of incipient inflammation, others with points of beginning suppuration at their centres, and others transformed into cysts containing a substance of the consistence of cream and of the colour of dregs of wine. In some of these last the cavity was as large as a pigeon's egg, and in two in the axilla it would have contained a hen's egg. The alteration had advanced furthest in the glands of the left axilla, less in those of the left groin, and still less in those of the right groin. The thoracic viscera were healthy except for a slight serous effusion in the right pleura; but the glands at the bifurcation of the trachea were enlarged to the size of a big fist. Internally they presented the various degrees of the inflammatory process observed in the other glands, though in a less advanced state; some presented points of suppuration, but the matter had not the lurid wine-dregs-colour seen in the glands of the external parts of the body. In the abdomen nothing anormal was found except incipient inflammation of the mesenteric glands. *Annali Universali di Medicina. Marzo*, 1840.

---

*On the State of the Blood in different Diseases.* By MM. Andral and Gavarret.

At the sitting of the Royal Academy of Sciences on the 27th of June last, M. Andral read in his own name and that of M. Gavarret a first memoir on the variation of the blood in connexion with diseases. This work is the result of examinations of the blood of 200 patients, and 360 bloodlettings. The proceeding followed by the authors is that which has been pointed out by MM. Prevost and Dumas.

In 1000 parts of blood, the fibrine has been found to vary in proportion from 1 to 10, the globules from 185 to 21, the solid matters of the serum from 104 to 57, the water from 915 to 725. It is very rarely that the different principles of the blood are simultaneously augmented or diminished under disease. Most frequently one or other of these principles is alone altered, but sometimes two are modified at the same time, and then it is in an inverse sense; thus, when the fibrine increases the globules diminish, and the contrary, and thus there results a remarkable change in the relations of quantity, which these principles should preserve with each other.

The diseases considered in relation to the changes which arise in the composition of the blood are divided into four classes. The first comprehends the diseases in which the fibrine is constantly augmented, as the phlegmasiæ. The second includes the diseases in which the fibrine is never augmented and often diminished, as the pyrexiæ. In the third class are the diseases in which there is a uniform diminution of the globules, as chlorosis. Lastly, in the fourth class are arranged the morbid states or fundamental alteration of the blood, in which the albumen of the serum is diminished, as in Bright's disease. But this is not all; the facts are not always thus simple, and it is often that many morbid acts, each of which causes a different modification in the blood, complicate it. For instance, when pneumonia seizes a chlorotic female, the blood still contains a small quantity of globules, but the quantity of fibrine is augmented. "We have seen," say the authors, "these results produced so frequently, that if we should find in the blood of any patient that there was more than five of fibrine, we should not fear to affirm by this alone, in that patient, a complication of one of the morbid states comprised in our first class, and on the other hand, where we found less than two of fibrine instead of more than five, we should deny the existence of such species of complication."

Besides diseases, losses of blood and privations of food notably modify the composition of the blood and add their influence to that of the disease. This fact is generally admitted; but the researches of MM. Andral and Gavarret have been directed to determine in what manner the composition of the blood is modified. Abstinence and losses of blood act principally by diminishing the globules. In the diseases in which the authors practised bloodlettings, these constantly rendered the number of globules less and less considerable in proportion as they were repeated. But, it is to be remarked, that the globules did not diminish from one bloodletting to the other in the same proportion in all the patients; in one the globules lose scarcely 2 or 3, but in another from 30 to 40. But while in all cases bloodlettings diminish the quantity of globules, the fibrine most frequently preserves its own proportion, being rarely diminished and sometimes increased. When the disease is of such a nature that an increase of the proportion of the fibrine is one of its necessary elements, this increase occurs notwithstanding the bloodletting and the diminution of the globules. In order that the losses of blood may have the power of diminishing the proportion of fibrine, they must be very considerable, and as in this case the globules are greatly diminished, it follows that all the solid elements of the blood are simultaneously diminished.

With this general exposition, M. Andral passes to the consideration of the facts relating to the alterations of the blood in the four classes of diseases which he has established.

FIRST CLASS. *Diseases in which the fibrine is augmented.* The augmentation of the proportions of this element has been established in two orders of diseases, namely, in the phlegmasiæ and in tubercular phthisis. The phlegmasiæ in which the blood has been examined are articular rheumatism, pneumonia, capillary bronchitis, pleuritis, tonsillitis, erysipelas, cystitis, acute suppuration of the lymphatic glands, and a furuncular eruption with fever. The blood has been examined in eighty-two individuals attacked by these diseases, and in 153 bloodlettings which were practised upon them. In all the cases where the diseases showed themselves in their acute form and were accompanied by fever, a very notable increase of fibrine was always found, varying in different diseases and different cases of the same disease. Thus, taking the proportion 3 as a normal medium for the fibrine, the following degrees of elevation were found: 1. In acute articular rheumatism, the mean quantity of fibrine varied between 7 and 8, the minimum being from 4 to 5, the maximum 10. 2. In pneumonia, the mean quantity of fibrine was the same as in the acute articular rheumatism, the maximum and minimum being the same. 3. In acute capillary bronchitis, the mean quantity of fibrine is not so great as in the two preceding diseases—it was between 6 and 7, the maximum being below 9. 4. In acute pleurisy, the mean quantity of fibrine again descends; it was from 5 to 6, the maximum not passing beyond 6. In acute peritonitis, the mean is also between 5 and 6, the maximum being 7. In the other diseases named above, the mean was about 5. In no case was the minimum below 4, and very rarely 5.

Thus in all the diseases called inflammatory, in which the blood has been examined, whatever their seat and degree of intensity, the fibrine has notably exceeded its normal proportion, the limits being 5 on one side and 10 on the other. But to support this rule there must be the double condition of acuteness and fever. For if the disease be primitively chronic or has become so, if fever has not existed or has disappeared, the fibrine ceases to be in excess in the blood. As to the state of acuteness, the increase in the proportion of fibrine is ruled by the intensity of the local symptoms and of the febrile disturbance. No acute inflammation produces more fibrine than pneumonia, and, after that, than acute articular rheumatism.

As the inflammation declines, the quantity of fibrine diminishes; if after recovering the patient relapses, the fibrine augments again. And if any acute inflammation supervenes during the course of any disease, it is immediately marked by an augmentation in the fibrine of the blood.

The globules in no case undergo increase in consequence of the state of phlegmasia; indeed in the commencement of affections of this class the globules rather appear to have diminished. In all the phlegmasiæ they decrease in proportion as the disease is prolonged, and the patients have been subjected to diet and bloodlettings. A great diminution in the proportion of the globules does not prevent inflammation from arising, increasing, or arriving at the fullest development; and on the other hand, a greatly increased proportion of the globules does not appear to favour its production. The observations of MM. Andral and Gavarret indeed show that inflammation is compatible with the most variable proportions of globules, as from 148 to 60.

In all these inflammatory diseases, the solid materials of the serum presented no alteration worthy of remark. The water varied from 771 to 840.

We have said that it is not only in the blood of individuals attacked with inflammation that an augmentation of fibrine occurs, but also in cases of pulmonary tubercles. At whatever period of phthisis the blood is examined, there is an augmentation of fibrine and a diminution of the globules; but the elevation of the first of these elements and the depression of the second are not equally marked in all the phases of the disease. When the tubercles are in the crude state, the fibrine is not considerably augmented, the mean proportion being about 4, and the diminution of the globules, though perfectly manifest, is not very great. When the tubercles begin to soften, the fibrine has a mean of 4½, and the globules continue to descend. When the lung is broken up by caverns, the fibrine has a mean of 5, and is sometimes elevated to nearly 6, but it never reaches the mean of pneumonia. When the disease has reduced the patient to the last degree of marasmus, the fibrine begins to decrease with the other solid elements of the blood, and descends below the normal proportion. In general, the greatest excess of fibrine in the blood of phthisical patients is shown when continued febrile disturbance is established.

The globules, in an inverse ratio with the fibrine, become less and less abundant in this period of phthisis. In the first stage of the disease they continue below 100, and never reach their mean quantity. In the second stage the proportion is generally depressed below 100, and in the third below 81. This diminution is doubtless very notable, but it is less than that of chlorosis.

The solid materials of the serum vary in phthisis between 64 and 98; the proportion 64 was afforded by a patient in whom the fibrine was only 2. The water is more abundant the more advanced the period of the disease. It varied from 784 to 845.

Second Class. *Diseases in which the fibrine is in its normal or in a diminished quantity, while the proportion of the globules is normal or augmented.* This class is divided into two orders: 1, the pyrexiæ or fevers; 2, many congestions and hemorrhages.

In *continued fevers*, the fibrine is never augmented and often diminished, sometimes even to a thousandth. The globules are never diminished before bloodletting, and frequently they are augmented to a degree exceeding 140. When simple continued fevers exist without appreciable inflammatory action, the modifications are those just stated. In one case, where all the symptoms characterizing inflammatory or *angéioténic* fever were present, the globules reached the enormous proportion of 185, and yet the fibrine maintained its normal proportion.

The authors apply the term *typhoid fever* to continued fevers, exhibiting at first an exanthematous affection and afterwards ulceration of the intestinal follicles. In consequence of the inflammatory condition of the intestinal alteration which forms the anatomical character of typhoid fever, it might be naturally supposed that the blood in this disease would, more or less, retain the qualities of the blood in the inflammations; but this is not the case, for whatever may be the intensity of the intestinal inflammation, the blood never takes these characters. In typhoid fever, at whatever period the blood is examined, and this examination has been made from the fourth to the twenty-first day, the fibrine is never found elevated above its normal proportion. It often preserves this point, but is frequently depressed below it, thus affording results directly opposite to those observed in the inflammations. Again, in inflammation the fibrine is augmented in a direct ratio with the intensity of the disease, but in the typhoid lesion, on the contrary, the fibrine diminishes in a direct ratio with the severity of the fever, and it may fall to below a thousandth. Of all diseases the typhoid fever is that in which the proportion of fibrine has been found to descend the lowest. As to the globules, whilst in inflammation they frequently have a slightly elevated proportion in the commencement of the disease, in typhoid fevers the tendency is reversed. Thus towards the eighth day, it is not rare to find the proportion of globules from 140 to 150, whilst in acute rheumatism and pneumonia at the same period, they are seldom elevated above 130. Now, at a similar period from the commencement of typhoid fever, notwithstanding bloodlettings and diet, we frequently find the globules maintain a proportion much above 130. And it is very remarkable that this elevated proportion has never been wanting or ceased to exist, that the fever has not been less severe or its progress more slow. We see, then, that typhoid fever is a morbid state infinitely more complex than an inflammation.

In the *eruptive fevers* (variola, varicella, rubeola, scarlatina,) the fibrine descends to 1 and never exceeded 4, and this maximum was only once observed. It is singular that in a disease, as variola, where the skin becomes the seat of an abundant suppuration, the blood, according to the law of inflammation, should not offer an augmentation of fibrine. But the cutaneous inflammation of variola, like the intestinal inflammation of typhoid fever, is nothing more than a simple element of a general predominant affection, the character of which is taken by the blood. The globules are considerably augmented in many cases of rubeola and scarlatina, to the proportion for instance of 146; on the contrary they are not augmented in a sensible manner in any one case of variola.

Cases of *intermittent fever* were rarely met with, and only negative results obtained.

In answer to the question whether "In inflammation is it the fever or the inflammation which produces an augmentation of fibrine in the blood?" the authors reply that "it is the inflammation, and that without the occurrence of the local lesion which constitutes this state, fever alone, whatever may be its intensity and duration, has not the effect of augmenting the quantity of fibrine contained in the blood." In the majority of *congestions and cerebral hemorrhages*, but not in all, the fibrine was found below its normal proportion, whilst the globules preserved or exceeded theirs; and this result was the more evident the nearer to the commencement of the disease the blood was examined.

THIRD CLASS. *Diseases in which the globules of the blood are diminished.* The memoir contains a number of facts relative to the morbid states of which this diminution is a constant character, as dropsies, the sequelæ of intermittent fevers, and the cachectic state presented by workers in lead, but we can only give the details on chlorosis. In this disease there is a first degree which is so slightly characterized by external signs, that at the first onset one might take the young women who are attacked for plethoric persons; but this is a false plethora which is in some degree announced by the state of the blood, as on analysis there are found less globules than in the normal state, though the diminution is as yet inconsiderable. In the advanced stages of the disease the globules are diminished to a degree not found in any other disease, unless the whole system has been exhausted by accidental or abundant hemorrhages. In one of these latter cases, cited in the preceding memoir, the proportion of the globules descended to 21; in chlorosis, the mean proportion has been found depressed from 127, its normal standing, to 38, but generally it is about 50.

After iron has been administered to chlorotic patients for a certain time, if we again examine the blood, we find the proportion of globules risen again. Thus in one case under the influence of this remedy, the proportion promptly mounted from 46 to 95. The other elements of the blood (except the water which increases in consequence of the diminution of the globules) remain completely unchanged. Thus the solid matters of the serum, varying from 94 to 75, are maintained in their normal proportion; and the fibrine does not descend with the progress of the disease, nor increase under the action of iron. This, however, applies to simple chlorosis, for if an inflammation complicate this affection, it will be marked by an increase in the proportions of fibrine.

FOURTH CLASS. *Diseases in which the albumen of the serum is diminished.* When the secretion of the kidney is so modified that the urine is mixed with a certain quantity of albumen, this principle is found in a less proportion in the blood. The authors found that the serum contained from 50 to 60 of albumen, instead of 72, the normal medium. In the different cases included in the fourth class, the other constituents of the blood presented accidental modifications in connexion with accidental causes. Thus in one of these cases, an acute inflammation supervening upon the principal disease greatly augmented the quantity of the fibrine; in another case, the prolonged privation of aliments greatly diminished the quantity of globules.

*Archives Générales de Médecine. Août et Septembre,* 1840.

*Acute Ovaritis induced by Uterine Injections.* By M. LEROY-D'ETIOLLES.

"THIS disease," says M. Leroy, "has been as yet very little studied. Having had occasion to observe two examples supervening after uterine injections, I have detected some characters which authors have not assigned to it. Most generally it follows labour, but in the two cases which I have observed the cause was very different. The most prominent symptoms were sudden pain and rapid development of a tumour on one side. The pain comes on in fits, it is very severe, and compared by the females to the pains of delivery; but, notwithstanding its great intensity, it is not augmented by pressure, differing in this respect from the pain of peritonitis. In no other disease have I observed tympanitis so considerable which contributes still further to the sufferings of the patients. It shows itself on the second or third day, and is combated by powerful purgatives."

In the two cases reported by M. Leroy, the injections were passed with moderate force into the uterus, by the aid of a gum elastic sound. In one the quantity of marsh-mallow water was ten drachms. In both cases the liquid had scarcely arrived in the uterine cavity, when the patients complained of an acute pain in one side. In one case the disease was regarded as a metro-peritonitis occasioned by the escape of the liquid into the peritoneum; but M. Leroy thought it was inflammation of the tube and ovary on the side corresponding to the pain. The symptoms he observed in the first case enabled him to recognize it in the latter. Neither of the patients died, but in both the sinking down of the ovarian tumours, which could be felt by the finger in the vagina, was accompanied by an abundant discharge by the anus.

*Gazette Médicale de Paris. Septembre* 5, 1840.

